# Mending the blood–brain barrier with photobiomodulation: insights from a humanized tricellular transwell model

**DOI:** 10.1113/JP291431

**Published:** 2026-04-25

**Authors:** Alexei Verkhratsky

**Affiliations:** ^1^ Faculty of Biology, Medicine and Health The University of Manchester Manchester UK

**Keywords:** astrocyte, blood‐brain barrier, endotheliocyte, pericyte

The blood–brain barrier (BBB) is a multicellular complex (often referred to as a neurovascular unit) which combines vascular (glyococalyx, brain endothelial cells, pericytes at the level of capillaries to which, at the level of arterioles and venules, smooth muscle cells are added) and neural (astrocytic endfeet, processes of oligodendroglia precursor cells and juxtavascular microglia) elements. The barrier per se is locked by tight junctions clasping brain endotheliocytes, whereas all other cellular elements are interlocked into a functional network supported my multiple communication routes, with astrocytes in particular contributing to the regulation of barrier maintenance and function (Verkhratsky & Pivoriunas, 2023).

The BBB is central to brain homeostasis, yet it remains a largely unmet therapeutic target. Across a wide spectrum of neurological conditions, including stroke, traumatic brain injury and neurodegeneration, BBB malfunction is not merely a consequence of disease but a driver of pathology. Hypoxia, in particular, represents a unifying insult that disrupts endothelial integrity, promotes oxidative stress and triggers inflammatory cascades that compromise barrier function. Despite its central role, there are currently no therapies that directly target the BBB to restore its integrity following hypoxic injury.

In this context, in this issue of *The Journal of Physiology*, the study by Domocos et al. (2026) provides a conceptual advance by demonstrating that photobiomodulation (PBM), a non‐invasive light‐based intervention, restores BBB function following hypoxic stress in a human multicellular model.

Another key strength of the study lies in the experimental platform itself. As already alluded to, the BBB is a multicellular complex, and the use of a humanized tricellular BBB model enables controlled interrogation of endothelial, astrocytic and pericytic responses within a physiologically relevant yet reductionist system. Such a platform is far more than a mechanistic tool; it provides a scalable and tractable system for molecular studies, functional interactions and drug discovery. The ability to isolate cell‐type‐specific responses while preserving key aspects of neurovascular organization makes this model particularly attractive for pharmaceutical applications, where there is a critical need for reliable human‐relevant systems to screen compounds targeting BBB function.

Within this framework, the study revealed the fundamental role of endothelial cells, which emerge as the primary drivers of both injury and postlesional recovery. Hypoxia drives a dominant endothelial response characterized by increased HIF‐1α signalling, metabolic disruption and upregulation of von Willebrand factor (vWF), all of which are reversed by PBM. The restoration of transendothelial electrical resistance (TEER), a measure of BBB integrity, provides a functional readout that anchors these molecular changes to BBB physiology. The use of automated TEER platforms such as CellZScope 2 allows continuous, non‐invasive monitoring of barrier integrity over time. This enables high‐resolution temporal analysis of BBB dynamics while minimizing experimental disturbance, thereby increasing reproducibility and reliability. From a translational perspective, such automated systems are particularly valuable for drug screening, where consistent and scalable functional readouts are essential.

The most conceptually relevant contribution of the study is the identification of vWF as a mechanistic node linking hypoxia to barrier malfunction. Traditionally viewed as a marker of endothelial activation and thrombosis, vWF is here positioned as an active regulator of BBB integrity, while the demonstration that small interfering RNA (siRNA)‐mediated knockdown of vWF partially recapitulates PBM‐induced barrier rescue shifts the interpretation from correlation to causation. This places thrombo‐inflammatory signalling at the centre of BBB breakdown, aligning with emerging evidence that endothelial activation, vWF release and coagulation–inflammation coupling are key drivers of neurovascular injury.

Beyond endothelial signalling, the study provides insight into how PBM modulates the neurogliovascular unit as a whole. The attenuation of HIF‐1α stabilization in brain endothelial cells, coupled with reduced production of reactive oxygen species in astrocytes and pericytes, suggests a coordinated yet cell‐type‐specific response. The PBM does not act as a general anti‐inflammatory stimulus, but rather reshapes stress responses in a targeted manner, with endothelial rescue occurring alongside modulation of neuroglial oxidative stress. By improving mitochondrial reserve, PBM may enable endothelial cells to maintain and repair barrier function under stress. This is particularly relevant given that endothelial decline in ageing and disease is increasingly understood as a failure of metabolic resilience rather than solely structural breakdown (Sweeney et al., 2018).

From a broader physiological perspective, the study reinforces the concept that BBB malfunction is an actively regulated state, going beyond disruption of tight junctions and reflecting dynamic changes in endothelial signalling, transport processes and cellular interactions. In parallel, astrocytic components of the neurovascular unit, including aquaporin‐4 (AQP4), contribute to water homeostasis and vascular coupling, and their spatial organization is increasingly recognized as critical for barrier function (Alhadidi et al., 2024). In recent work from the same group, AQP4 has been shown to undergo dynamic subcellular relocalization, further emphasizing that membrane organization rather than expression alone governs function (Salman et al., 2022).

The pathophysiological implications of this work are significant. Hypoxia is a common denominator across acute and chronic neurological disorders, yet therapeutic strategies have largely focused on neuronal protection. This study shifts the focus towards the vasculature, suggesting that restoring endothelial function may be an effective approach to improve overall brain health. The identification of vWF and thrombo‐inflammatory pathways as modulators of BBB integrity similarly opens new avenues for intervention.

Equally important is the translational potential of the platform itself. The combination of human multicellular models with automated, non‐invasive functional readouts provides a framework for systematic evaluation of BBB‐targeted therapies. Such systems could be readily adapted for high‐content screening of candidate compounds, bridging the gap between basic mechanistic studies and pharmaceutical development.

In summary, Domocos and colleagues provide compelling evidence that PBM can restore BBB function after hypoxic injury through modulation of endothelial thrombo‐inflammatory signalling. By identifying vWF as a key mediator and integrating metabolic, inflammatory and functional readouts, the study advances both our understanding of BBB physiology and the development of new therapeutic strategies. At the same time, it highlights the growing importance of human‐relevant, quantitatively robust model systems in enabling both mechanistic discovery and translational progress.

## Additional information

### Competing interests

No competing interests are declared.

### Author contributions

A. V.: Conception or design of the work; Drafting the work or revising it critically for important intellectual content; Final approval of the version to be published; Agreement to be accountable for all aspects of the work.

### Funding

None.

## Supporting information


Peer Review History
