# Supplementary material for: Mending the blood–brain barrier with photobiomodulation: insights from a humanized tricellular transwell model
Source: J Physiol. 2026 Apr 25;604(14):5690–1. doi: 10.1113/JP291431 (PMC13370713; doi:10.1113/JP291431)
Supplement: Supplementary file 1 — Peer Review History [file TJP-604-5690-s001.pdf]

The Journal of Physiology

Mending BBB with photobiomodulation: insides from humanised tricellular transwell model

Alexei Verkhratsky
DOI: 10.1113/JP291431

Corresponding author(s): Alexei Verkhratsky (alexej.verkhratsky@manchester.ac.uk)

The following individual(s) involved in review of this submission have agreed to reveal their identity: Mootaz M. Salman (Referee #1)

Review Timeline:

Submission Date:	25-Mar-2026
Accepted:	14-Apr-2026

Senior Editor: Nathan Schoppa

Reviewing Editor: Valentina Mosienko

Transaction Report:

(Note: With the exception of the correction of typographical or spelling errors that could be a source of ambiguity, letters and reports are not edited. Depending on transfer agreements, referee reports obtained elsewhere may or may not be included in this compilation. Referee reports are anonymous unless the Referee chooses to sign their reports.)

Dear Professor Verkhratsky,

Re: JP-P-2026-291431 "Mending BBB with photobiomodulation: insides from humanised tricellular transwell model" by Alexei Verkhratsky

We are pleased to tell you that your paper has been accepted for publication in The Journal of Physiology.

The last Word (or similar) version of the manuscript provided will be used by the Production Editor to prepare your proof. When this is ready you will receive an email containing a link to Wiley's Online Proofing System. The proof should be thoroughly checked and corrected as promptly as possible.

Authors should note that it is too late at this point to offer corrections prior to proofreading. Major corrections at proof stage, such as changes to figures, will be referred to the Editors for approval before they can be incorporated. Only minor changes, such as to style and consistency, should be made at proof stage. Changes that need to be made after proof stage will usually require a formal correction notice.
All queries at proof stage should be sent to: TJP@wiley.com.

Following copyediting and typesetting the accepted version will be published online.

Please note that Perspective articles are not typically covered by institutional open access agreements with our publisher, Wiley. Wiley do not offer article processing charge (APC) discounts for smaller article types in hybrid subscription journals, meaning that if you wish for your Perspective to be published Open Access, you will have to pay the full APC. As such, we recommend authors publish Perspectives 'behind the paywall', where they will become freely accessible after a 12-month embargo (i.e. please select the NON open access option via 'Wiley Authors' during proofing).

Should you wish to pay for Open Access, you will be able to place an order by logging into Wiley Author services.

• You can check if your funder or institution has a Wiley Open Access Account here:
https://authors.wiley.com/author-resources/Journal-Authors/open-access/author-compliance-tool.html

Yours sincerely,

Nathan Schoppa
Senior Editor
The Journal of Physiology

IMPORTANT POINTS TO NOTE FOLLOWING ACCEPTANCE OF YOUR PAPER:

• IMPORTANT NOTICE ABOUT OPEN ACCESS: To assist authors whose funding agencies mandate immediate public access to published research findings, The Journal of Physiology allows authors to pay an Open Access (OA) fee to have their papers made freely available immediately on publication.

The Corresponding Author will receive an email from Wiley with details on how to register or log in to Wiley Authors where you will be able to place an order.

• You can check if your funder or institution has a Wiley Open Access Account here: https://authors.wiley.com/author-resources/Journal-Authors/open-access/author-compliance-tool.html

• TRANSPARENT PEER REVIEW POLICY: To improve the transparency of its peer review process, The Journal of Physiology publishes online as supporting information the peer review history of all articles accepted for publication. Readers will have access to decision letters, including Editors' comments and referee reports, for each version of the manuscript as well as any author responses to peer review comments. Referees can decide whether or not they wish to be named on the peer review history document.

• You can help your research get the attention it deserves! Check out Wiley's free Promotion Guide for best-practice recommendations for promoting your work at: www.wileyauthors.com/eeo/guide. You can learn more about Wiley Editing Services which offers professional video, design, and writing services to create shareable video abstracts, infographics, conference posters, lay summaries, and research news stories for your research at: www.wileyauthors.com/eeo/promotion.

• If you would like to receive our 'Research Roundup', a monthly newsletter highlighting the cutting-edge research published in The Physiological Society's family of journals (The Journal of Physiology, Experimental Physiology, Physiological Reports, The Journal of Nutritional Physiology and The Journal of Precision Medicine: Health and Disease), please click this link, fill in your name and email address and select 'Research Roundup':
https://www.physoc.org/journals-and-media/membernews

EDITOR COMMENTS

Reviewing Editor:

Many thanks for writing an excellent perspective on the Domocos et al. study recently accepted for publication in the Journal of Physiology. We greatly appreciate you taking the time to highlight for our readers the strengths and impact of this work.

Senior Editor:

Thank you for writing this Perspective on the research article by Domocos and co-workers. The reviewer, one of the authors of that article, considered your manuscript elegant and insightful, and the editors agree that it does an excellent job of explaining the impact of the work at different levels. We very much appreciate you taking the time to highlight the work for the general readership of The Journal of Physiology.

REFEREE COMMENTS

Referee #1:

The Perspective accurately captures the central findings and conceptual advance of our study. In particular, it appropriately highlights the restoration of barrier integrity following hypoxia, the central role of endothelial signalling, and the identification of von Willebrand factor (vWF) as a mechanistic mediator of BBB dysfunction and recovery. The discussion of photobiomodulation (PBM) as a targeted modulator of endothelial stress responses is well aligned with our data and interpretation.

It also correctly emphasises the importance of using a human multicellular BBB model and the relevance of functional readouts such as TEER, as well as the broader implications for therapeutic development and drug screening. We agree with the interpretation that our findings move beyond a general cytoprotective view of PBM towards a more mechanistically defined effect on thrombo-inflammatory signalling pathways.

In conclusion, this is an elegant and well-balanced Perspective that provides an accurate and insightful synthesis of our work and its broader significance.
